# Intraspecific differences in seed dispersal caused by differences in social rank and mediated by food availability

**DOI:** 10.1038/s41598-020-58381-0

**Published:** 2020-01-30

**Authors:** Yamato Tsuji, Ahimsa Campos-Arceiz, Soumya Prasad, Shumpei Kitamura, Kim R. McConkey

**Affiliations:** 1 Primate Research Institute, Kyoto University, 41-2 Kanrin, Inuyama, Aichi 484-8506 Japan; 2 School of Environmental and Geographical Sciences, The University of Nottingham Malaysia, Jalan Broga, Semenyih, Selangor 43500 Malaysia; 3 National Institute of Advanced Studies, Indian Institute of Science Campus, Bengaluru, 5600012 India; 4 Ishikawa Prefectural University, 1-308, Suematsu, Nonoichi, Ishikawa, 921-8836 Japan

**Keywords:** Behavioural ecology, Forest ecology

## Abstract

We use individual-based information on the behavior of wild female Japanese macaques in two consecutive years with different food availability (nut-rich vs. nut-poor) to test effects of dominance rank and nut fruiting on seed dispersal parameters. We predicted that social rank would affect dispersal (1) quantity, (2) quality, (3) species richness, and (4) percentage of berries in the diet in the nut-poor year, while these differences would disappear in the nut-rich year. We found seeds of nine fleshy-fruited plant species in the feces of the monkeys. The frequency of seed occurrence for two plant species (*Viburnum dilatatum* and *Rosa multiflora*) showed an interaction between dominance ranks and years; in the nut-poor year *V. dilatatum* seeds were more abundant among dominant females and *R. multiflora* among subordinates, while such inter-rank differences disappeared in the nut-rich year. Similarly, the intact ratio of *V. dilatatum* seeds was lower for dominants in the nut-poor year, while inter-rank variations disappeared in the nut-rich year. Finally, percentage of berries in diet and seed richness showed no inter-annual nor inter-rank variations. Our study highlights that differences in individuals’ social rank lead to within-group variation in seed dispersal services and that these differences are dependent on nut availability.

## Introduction

Seed dispersal interactions that occur among animals and plants are inherently complex. Part of this complexity arises from the broad communities of interacting plant and animal species and the context-dependency of these interactions, such as temporal changes in food availability for animals^1–4^. Intraspecific variation within animal and plant populations is another key source of variability for seed dispersal outcomes^5–7^. Although the ecological effect of intraspecific variability has rarely been taken into account, it has been documented to influence seed dispersal outcomes across almost all frugivore groups, including insects^8^, other invertebrates^9^, fish^10,11^, reptiles^12,13^, birds^14,15^ and mammals^16–19^ and for plants as well^5^.

Sources of intraspecific variability within animal populations might result from sex differences, ontogenetic shifts in diet or behaviour, individual specialization or behavioral syndromes^7^. An individual’s social rank within a primate group determines its access to favored resources and can influence its handling behaviour and movement patterns^20^, which are important behaviours associated with seed dispersal^21^.

While social rank and food availability have been highlighted as independent sources of variation that influence seed dispersal outcomes^2,3,7^, it is likely that their impacts frequently interact. Variance in the spatial and temporal availability of fruit^2,22–24^ will influence competitive interactions within animal populations and are a potential key driver of intraspecific variability in the outcomes of seed dispersal. Hence, it is essential to develop an understanding of the joint impacts of food availability and social rank.

Japanese macaques (*Macaca fuscata*) are omnivorous primates endemic to the Japanese archipelago^25,26^. Among fruits, they prefer nuts to berries due to their higher fat contents^27^. Japanese macaques are also known to be important seed dispersers^28,29^. Intraspecific variation in seed dispersal is likely to be caused by high competition over food that triggers inter-rank differences in food access^27,30,31^ and feeding behaviour^32^. Severe competition over food resources (e.g., clumped distribution of food resources, and lower availability) forced subordinates to use non-preferred feeding patches, to process food faster, to increase the number of food items consumed, to extend feeding time, and to reduce the time allocated to rest and social activities^30,32,33^. The Japanese macaques inhabit a highly seasonal environment in which both temperature and resources fluctuate throughout the year^34^. In this environment, the masting of nuts, which are favored by the macaques, can influence the intensity of inter-rank interactions. On Kinkazan Island, where nuts of species such as *Fagus crenata* (Fagaceae) and *Zelkova serrata* (Ulmaceae) are preferred food items for the macaques, an inter-rank variation in feeding behavior between a nut-poor year (2004) and a masting nut-rich year (2005) has been reported. In 2004, only dominants obtained enough energy from nuts, while in 2005 all the group members could feed on nuts regardless of their social rank^27^. If inter-rank variation in feeding behavior is modified by variations in food availability, the characteristics of endozoochory shown by dominants and subordinates should also vary accordingly (e.g. inter-annually in the case of Kinkazan Island).

If individual differences are to have a strong impact on seed dispersal, they must impact the behaviours that drive seed dispersal outcomes^7^. Using data from macaques on Kinkazan over nut-poor and nut-rich years, we investigated whether key seed dispersal parameters^35^ differ across social-ranks (high, middle, and low). Specifically, we evaluated the parameters of 1) quantity, 2) quality, 3) species richness, and 4) proportion of berries, as a trait of dispersed propagules^7^. Because Japanese macaques generally move as a group, we did not consider effects on movement distances, another parameter of seed dispersal. We also determined the influence of nut-availability (rich and poor) on the behaviour of animals with different social ranks. We predicted that high competition for preferred foods (nuts) in low resource years would cause differences in the seed dispersal parameters measured among individuals of different social ranks. These differences should not be present in nut rich years.

## Results

### Seed dispersal quantity

During the study period, we collected 99 fresh fecal samples in total (n = 56 in 2004 and 43 in 2005), from which we identified 9,013 seeds from the following nine fleshy-fruit plant species: *Viburnum dilatatum* (Adoxaceae), *Rosa multiflora* (Rosaceae), *Vitis flexuosa* (Vitaceae), *Cornus kousa* (Cornaceae), *Viscum album* (Santalaceae), *Pourthiaea villosa* (Rosaceae), *Ilex macropoda* (Aquifoliaceae), *Swida macrophylla* (Cornaceae), and *Malus tschonoskii* (Rosaceae; Table 1). Seed appearance ratios for all species combined were 0.96 (54 out of 56) in 2004 and 0.91 (39 of 43) in 2005 and showed no significant variation between years and among dominance ranks (χ^2^ = 0.14, df = 5, p = 1.000). At the plant species level, however, there was significant inter-annual and inter-rank variation in the appearance ratios of *R. multiflora* (42 of 56 in 2004 vs. 6 of 43 in 2005; χ^2^ = 14.31, df = 5, p = 0.014) and *V. flexuosa* (1 of 56 in 2004 vs. 19 of 43 in 2005; χ^2^ = 18.68, df = 5, p = 0.002).

**Table 1:** Obtained values and results of statistical tests on characteristics of seed dispersal by wild Japanese macaques on Kinkazan Island, northern Japan.

Variables	Obtained values						Explanatory variables					
	2004			2005			Main effects				Interaction	
	High (n = 12)	Middle (n = 19)	Low (n = 25)	High (n = 17)	Middle (n = 11)	Low (n = 15)	Rank		Year		Rank × Year	
**a) Seed appearance ratio**												
All species	0.92	1.00	0.96	0.94	0.82	0.93	χ^2^ = 0.00	p = 1.000	χ^2^ = 0.00	p = 0.999	χ^2^ = 0.14	p = 1.000
*Viburnum dilatatum*	0.42	0.63	0.68	0.82	0.64	0.60	χ^2^ = 0.01	p = 0.996	χ^2^ = 0.08	p = 0.653	χ^2^ = 12.06	p = 0.944
*Rosa multiflora*	0.75	0.74	0.76	0.18	0.18	0.07	χ^2^ = 0.32	p = 0.852	χ^2^ = 12.56	p < 0.001***	χ^2^ = 14.31	p = 0.014*
*Vitis flexuosa*	0.08	0.00	0.00	0.53	0.36	0.40	χ^2^ = 3.26	p = 0.196	χ^2^ = 15.72	p < 0.001***	χ^2^ = 18.68	p = 0.002**
*Viscum album*	0.67	0.21	0.16	0.00	0.00	0.00	χ^2^ = 5.16	p = 0.076	N/A		N/A	
*Cornus kousa*	0.00	0.00	0.00	0.12	0.09	0.20	χ^2^ = 0.55	p = 0.759	N/A		N/A	
*Ilex macropoda*	0.25	0.21	0.12	0.00	0.00	0.00	χ^2^ = 0.79	p = 0.673	N/A		N/A	
*Pourthiaea villosa*	0.08	0.05	0.04	0.29	0.09	0.13	χ^2^ = 2.92	p = 0.231	χ^2^ = 2.34	p = 0.105	χ^2^ = 5.78	p = 0.328
*Swida macrophylla*	0.00	0.00	0.00	0.06	0.09	0.07	χ^2^ = 2.57	p = 0.765	N/A		N/A	
*Malus tschonoskii*	0.00	0.00	0.04	0.00	0.00	0.07	χ^2^ = 2.87	p = 0.239	χ^2^ = 0.00	p = 1.000	χ^2^ = 3.16	p = 0.675
**b) Number of seeds**												
All species	94.1 ± 188.4	100.1 ± 171.6	142.4 ± 231.1	89.4 ± 146.9	50.1 ± 53.1	28.7 ± 31.3	z = 16.66	p < 0.001***	z = 23.00	p < 0.001***	z = −19.83	p < 0.001***
*Viburnum dilatatum*	143.2 ± 248.7	25.8 ± 30.2	45.2 ± 52.1	75.5 ± 121.4	73.6 ± 47.1	35.4 ± 36.5	z = −4.30	p < 0.001***	z = 2.35	p = 0.019*	z = 3.90	p < 0.001***
*Rosa multiflora*	34.2 ± 35.1	106.5 ± 190.7	145.9 ± 249.3	64.7 ± 71.2	6.5 ± 3.5	1.0 ± 0.0	z = 11.90	p < 0.001***	z = 12.95	p < 0.001***	z = −11.47	p < 0.001***
*Vitis flexuosa*	10.0 ± 0.0	—	—	14.0 ± 13.7	2.8 ± 3.0	13.7 ± 14.0	z = 0.75	p = 0.455	N/A		N/A	
*Viscum album*	10.6 ± 10.8	18.3 ± 12.6	2.0 ± 1.2	—	—	—	z = −2.17	p = 0.030*	N/A		N/A	
*Cornus kousa*	—	—	—	11.5 ± 4.5	2.0 ± 0.0	7.0 ± 7.1	z = 4.29	p < 0.001***	N/A		N/A	
*Ilex macropoda*	2.7 ± 2.4	6.3 ± 4.6	3.0 ± 2.8	—	—	—	z = 0.92	p = 0.357	N/A		N/A	
*Pourthiaea villosa*	1.0 ± 0.0	1.0 ± 0.0	1.0 ± 0.0	10.0 ± 8.2	6.0 ± 0.0	1.0 ± 0.0	N/A		N/A		N/A	
*Swida macrophylla*	—	—	—	4.0 ± 0.0	3.0 ± 0.0	1.0 ± 0.0	N/A		N/A		N/A	
*Malus tschonoskii*	—	—	1.0 ± 0.0	—	—	8.0 ± 0.0	N/A		N/A		N/A	
**c) Intact ratio**												
All species	0.85 ± 0.21	0.88 ± 0.18	0.94 ± 0.09	0.98 ± 0.06	1.00 ± 0.00	0.99 ± 0.02	z = 6.31	p < 0.001***	z = 6.05	p < 0.001***	z = −6.19	p < 0.001***
*Viburnum dilatatum*	0.83 ± 0.34	0.91 ± 0.28	0.97 ± 0.07	1.00 ± 0.00	1.00 ± 0.00	1.00 ± 0.00	z = 3.66	p < 0.001***	z = 5.89	p < 0.001***	z = −3.45	p < 0.001***
*Rosa multiflora*	0.86 ± 0.16	0.84 ± 0.20	0.91 ± 0.11	1.00 ± 0.00	1.00 ± 0.00	1.00 ± 0.00	z = 0.19	p = 0.847	z = −0.10	p = 0.922	z = −0.12	p = 0.904
*Vitis flexuosa*	1.00 ± 0.00	−	−	1.00 ± 0.00	1.00 ± 0.00	1.00 ± 0.01	z = 0.02	p = 0.987	N/A		N/A	
*Viscum album*	0.91 ± 0.25	1.00 ± 0.00	0.88 ± 0.22	−	−	−	z = −0.00	p = 0.350	N/A		N/A	
*Cornus kousa*	—	—	—	1.00 ± 0.00	1.00 ± 0.00	1.00 ± 0.00	z = 0.00	p = 1.000	N/A		N/A	
*Ilex macropoda*	0.94 ± 0.08	0.88 ± 0.22	1.00 ± 0.00	—	—	—	z = 0.00	p = 1.000	N/A		N/A	
*Pourthiaea villosa*	1.00 ± 0.00	1.00 ± 0.00	1.00 ± 0.00	0.76 ± 0.15	1.00 ± 0.00	0.50 ± 0.50	N/A		N/A		N/A	
*Swida macrophylla*	—	—	—	0.50 ± 0.00	0.00 ± 0.00	1.00 ± 0.00	N/A		N/A		N/A	
*Malus tschonoskii*	—	—	1.00 ± 0.00	—	—	1.00 ± 0.00	N/A		N/A		N/A	
d) Seed species richness	3.00 ± 1.00	2.53 ± 1.23	2.20 ± 1.06	2.41 ± 1.09	2.18 ± 1.03	1.87 ± 1.09	z = −0.55	p = 0.585	z = −0.81	p = 0.417	z = 0.49	p = 0.622

*p < 0.05, **p < 0.01, ***p < 0.001. We could not test the significance of number of seeds and intact ratio for *Swida macrophylla*, *Malus tschonoskii*, and *Pourthiaea villosa* due to their small sample sizes.

**Figure 1:**
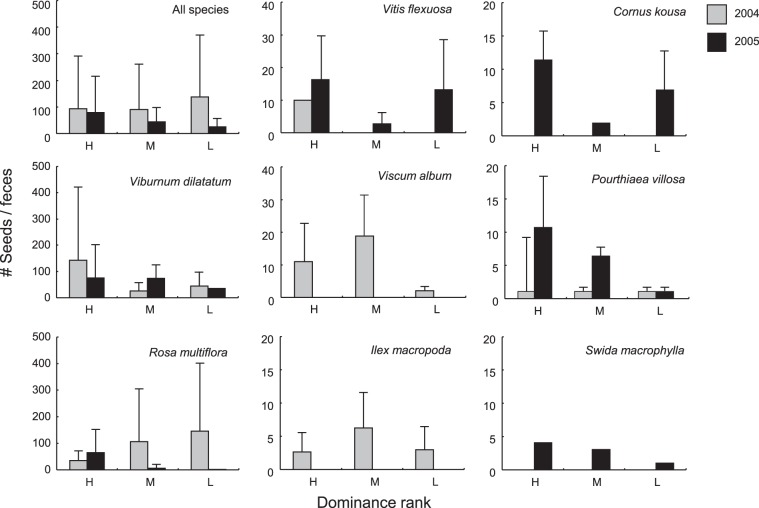
Inter-annual and inter-rank variation in mean (±SD) seed number per single feces collected in fall (October and November). H: high-rank, M: middle-rank, L: low-rank.

The total number (mean ± SD) of seeds per fecal sample ranged from 29 ± 31 (low rank individuals in 2005) to 142 ± 231 (low rank individuals in 2004; Table 1). Seeds of *V. album* and *I. macropoda* appeared only in 2004, while the seeds of *C. kousa* and *S. macrophylla* appeared only in 2005, likely due to inter-yearly differences in their availability (Fig. 1). The seeds of *V. flexuosa* and *M. tschonoskii* were found exclusively in the feces of specific dominance rank females (e.g. *V. flexuosa* appeared only in feces of high-rank females in 2004; Fig. 1). Our analyses were therefore restricted for these species. Further, for three species (*P. villosa*, *S. macrophylla*, and *M. tschonoskii*) we could not conduct statistical analyses due to small sample sizes.

In the number of seeds per fecal sample we found a significant year-rank interaction for all species together, *V. dilatatum*, and *R. multiflora* (Table 1 and Fig. 1) – the number of seeds of all species together and *V. dilatatum* seeds were greater in feces of dominants (all species: z = −19.83, p < 0.001, *V. dilatatum*: z = 3.90, p < 0.001) while the number of *R. multiflora* seeds was larger in the feces of subordinates in 2004 (z = −11.47, p < 0.001) but not in 2005, when there were no inter-rank variations (Fig. 1). For *V. album* (z = −2.17, p = 0.030, H < M > L) and *C. kousa* (z = 4.30, p < 0.001, H > M < L), we also found inter-rank differences in seed numbers per fecal sample (Fig. 1).

### Seed dispersal quality

**Figure 2:**
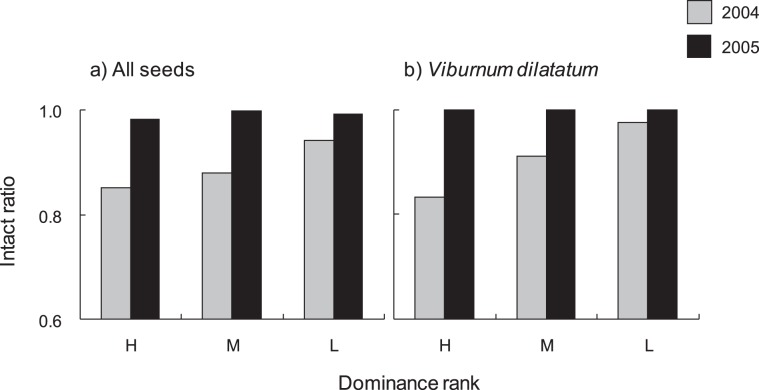
Inter-annual and inter-rank variation in mean intact ratio of seeds of the Japanese macaques for the (**a**) all plant species and (**b**) *Viburnum dilatatum* in fall (October and November). The intact ratio is obtained by dividing total number of intact seeds by total number of seeds in a given fecal sample. H: high-rank, M: middle-rank, L: low-rank.

The seed intact ratio ranged from 0.85 to 1.00 (Table 1). The interaction between year and dominance rank was significant for the seeds of all species lumped together (z = −6.19, p < 0.001) and of *V. dilatatum* (z = −3.5, p < 0.001). In 2004, seed intact ratios were lower in dominants than in subordinates, while inter-rank variations disappeared in 2005 (Fig. 2).

### Species richness

The mean seed species richness per fecal sample ranged from 1.9 to 3.0 species across dominance groups and years (Table 1). Neither year (z = −0.81, p = 0.417), dominance rank (z = −0.55, p = 0.585), nor their interaction (z = 0.49, p = 0.622) had any significant effect on seed species richness (Table 1).

### Traits of dispersed propagules (proportion of berries)

**Figure 3:**
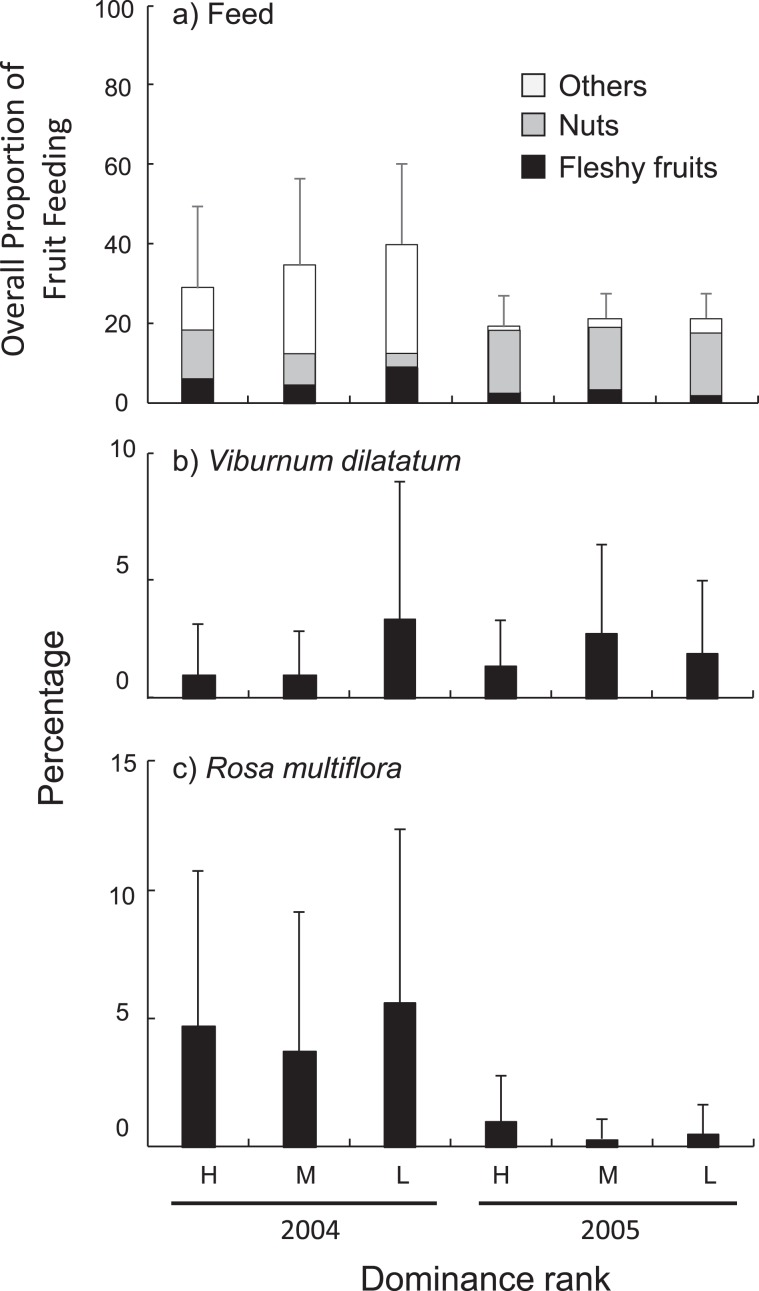
Inter-annual and inter-rank difference in percentage of (**a**) feeding (filled part and grey part represent fleshy fruits and nuts, respectively) of the adult female Japanese macaques on Kinkazan Island, northern Japan. For the fleshy-fruit feeding, we also show percentage of the main two species (**b**) *Viburnum dilatatum* and (**c**) *Rosa multiflora*. H: high-rank, M: middle-rank, L: low-rank.

The percentage of total feeding (across all items) differed significantly between years (2004 > 2005; z = −4.92, p < 0.001) and among dominance ranks (high < middle < low; high vs. middle: z = −2.62, p = 0.009; high vs low: z = −2.17, p = 0.030; Fig. 3a). The percentage of nut feeding also varied between years (2004 < 2005; z = 3.67, p < 0.001) and among dominance ranks (high vs. middle: z = −0.24, p = 0.814; high vs low: z = −2.11, p = 0.035; Fig. 3a). The percentage of fleshy-fruit feeding, however, did not vary between years (z = −0.19, p = 0.848, Fig. 3a) nor among dominance ranks (high vs. middle: z = −0.23, p = 0.821; high vs low: z = 1.65, p = 0.098). We conducted the analyses for two fleshy-fruit species: for *V. dilatatum* we found no significant difference between years (z = 0.81, p = 0.419, Fig. 3b) and among dominance ranks (high vs middle: z = −0.04, p = 0.972; high vs low: z = 1.77, p = 0.077); for *R. multiflora*, we found a significant year-rank interaction (high rank in 2004 vs. low in 2005: z = −2.00, p = 0.045; and high in 2004 vs. middle in 2005: z = −2.51, p = 0.012). The percentage of *R. multiflora* feeding by low-ranking females was higher than by high and middle-ranking females in 2004, while such inter-rank differences disappeared in 2005 (Fig. 3c).

## Discussion

**Figure 4:**
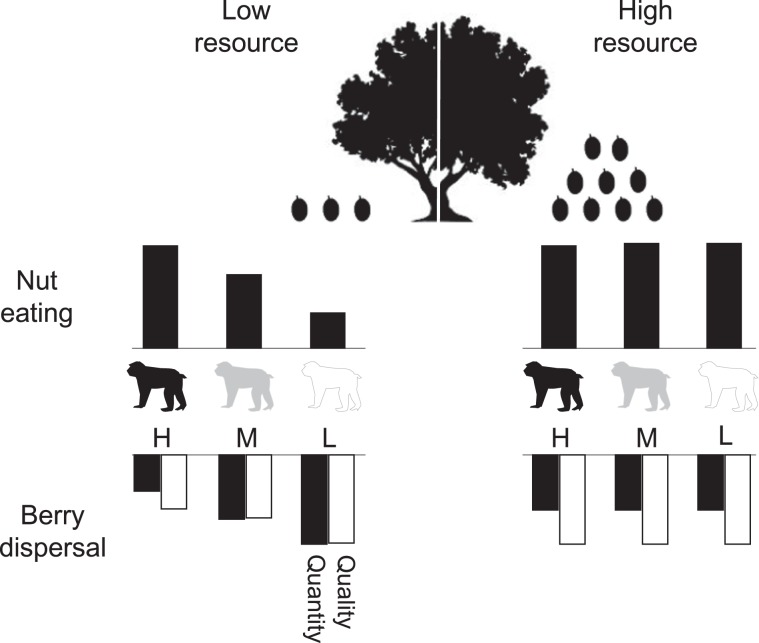
A schematic chart of the effects of social rank and nut fruiting (nut-poor year in 2004 and nut-rich year in 2005) on (1) nut eating, (2) quantitative and (3) qualitative parameters of berry dispersal, based on results of this study. H: high-rank, M: middle-rank, and L: low-rank. Images were drawn by FLOP DESIGN (Masashi Kato).

Seed dispersal services performed by Japanese macaques were related to resource abundance, which differentially influenced the roles played by individuals of different social ranks. In years when resource abundance was low, high-ranking individuals dominated preferred resources (nuts), which forced low ranking individuals to consume, and disperse, berries. More berry seeds were also chewed up by the high ranking individuals during this year, probably to maximize the nutritional value as has been observed in other monkey species^16,36^. Low ranking individuals were higher quality seed dispersers as they had lower rates of seed mastication, perhaps as a result of having to swallow foods quickly to avoid conflict^27,33^. Conversely, in the nut-rich year, these differences among individuals of different social ranks disappeared, and the overall impact on seed dispersal was slightly reduced in quantitative terms (eating fewer berries) but increased in qualitative terms (due to lower rates of seed mastication for berries). Hence, Japanese macaque’s seed dispersal behaviour was impacted by both social rank and nut availability as well as the interaction between them (Fig. 4).

Two conditions are necessary for individual variation within populations to exert a strong influence on seed dispersal^7^. First, the differences among individuals should have a strong influence on seed dispersal outcomes. Among the four seed dispersal parameters we measured, we found three significant intraspecific differences (quality, quantity, and propagule-identity) in the nut poor year. Low and middle ranking individuals were more effective dispersers than high ranking individuals, because they dispersed a higher quantity of seeds, and were also higher quality dispersers, due to a reduced rate of seed mastication and a higher consumption of berries. However, individuals of different ranks showed no difference in species richness dispersed. While this intraspecific variation was not found in the high resource year, masting events in northern Japan happen around every 5–7 years^37,38^, and in most years there is competition over preferred resources^27,29^. Hence, intraspecific differences among individuals of different social ranks probably occur in most years for the Japanese macaques.

Troop size is another variable that can influence seed dispersal outcomes. The troop size (N = 35) of the studied group is average for this species^39^. However, in other populations, particularly on the mainland, larger troops sometimes occur and this will increase the number of individuals in each social rank and potentially vary the ratio of individuals across ranks. The relative contribution of specific ranked animals on dispersal could be much greater when the ratio of high to low ranking is different.

Under scenarios of anthropogenic disturbance, this intraspecific variation we observed could have important consequences for seed dispersal at the population level for Japanese macaques^40,41^. Group sizes can be reduced due to direct mortality (e.g., conflict with farmers^42^), or due to habitat or resource change which causes large groups to either split or adjust to the reduction in resources^43^. Under these scenarios, competition for resources may be reduced among social ranks, or there may be fewer low-ranking individuals, negatively shifting the population averages for most seed dispersal parameters we measured. Competition for resources may also be reduced under scenarios of provisioning by humans because it increases resource abundance, further reducing differences among social ranks^41,44^. Conversely, a population increase – perhaps due to more individuals being forced into a smaller area – would increase competition and the differences in seed dispersal outcomes across social ranks. Overall this could lead to a higher population-level seed dispersal effectiveness.

The importance of density-dependence is well established in the seed dispersal literature, mainly focusing on the effects of temporal changes in food availability^2,3,45^. We and recent studies^7,46^ have found that the temporal availability of fruit influences frugivore choices, which in turn causes intraspecific variation in seed dispersal parameters. Intraspecific variability in both plants and animals does not simply add noise to systems, but alters dispersal processes and patterns with consequences for plant demography, communities, evolution and responses to anthropogenic changes; by reducing this variability to an average value, important details on the seed dispersal capacity and resilience of animals has been lost^5–7,46^. Individuals within a population respond to fruit abundance, which might alter the extent and direction of intraspecific variation within populations. Hence, more research into understanding these inter-relationships are required, particularly as the source of context-change is increasingly a form of anthropogenic disturbance^5,41^.

So far, the detailed intra-group data that can provide a wealth of information on social interactions and behavior, has been collected by primatologists, as it is difficult or impossible to collect similar data for many other animal groups^47^. This information could be used to explore the relationships between intraspecific variation and context dependency in seed dispersal across a broader range of taxa and habitats. Because context-dependency can alter competitive relationships under a variety of scenarios^20^ and, potentially, in any animal taxa, it is likely that many other sources of intraspecific variation can also be context-dependent; for example, differences in dispersal due to body size or sex could be a result of dominance-hierarchies in some cases. Japanese macaques are known to leave the natal troop and remain as solitary individuals or move into different troops^48^. This means that the interspecific differences in seed dispersal that we describe could be more extreme if males were considered. It is of particular urgency to explore the influence that disturbance contexts can have on intra-specific variability and seed dispersal effectiveness.

## Methods

### Ethics statement

Our field study did not involve endangered or protected animal and plant species. The research methodology complied with protocols approved by the guidelines (Guide for the Care and Use of Laboratory Primates, Third Edition) of the Primate Research Institute, Kyoto University, Japan, and the legal requirements of Japan.

### Study site and subjects

Kinkazan Island (38.3°N, 141.6°E) is located 700 m off the Oshika Peninsula, in northern Japan. The total area of the island is ca. 9.6 km^2^, and the highest peak is 450 m a.s.l. The monthly mean air temperature on the island ranges from 2.5 °C in February to 22.3 °C in August. Deciduous forests of *Fagus crenata* (Fagaceae) dominate the higher elevations (>150 m), whereas a mixture of deciduous forests of *Carpinus* spp. (*C. tschonoskii* and *C. laxiflora*, Betulaceae) and coniferous forests of *Abies firma* (Pinaceae) cover the lower elevations (<150 m) on the island^23,27^.

Behavioral observation and fecal sample collection took place in two months in both 2004 and 2005. During this period, approximately 200–250 Japanese macaques belonging to six troops inhabited the island^49^. Our study subjects were the adult (>5 yr) females of one of these troops called Troop A. This troop has been habituated to observation at close proximity (<10 m) by researchers since 1982. During the study period, the size of Troop A varied from 29 to 39 individuals, including 2–5 adult males, 14–17 adult females, 8–9 juveniles (1–5 yr), and 1–12 infants (<1 yr), and we had good knowledge of the maternal kinship and dominance ranks of the 17 adult females^27^.

### Fecal sample collection and analysis

During the study period, we collected fresh fecal samples from each focal animal during our observations. Each sample was rinsed through 0.5 mm sieves under fresh water. We picked up all seeds from the fecal samples, identified seeds to the species level, and counted the number of seeds of each species. Seed identification was based on our previous work^29,50^.

We analyzed seed data by year (2004 vs. 2005) and the individual macaque dominance rank (high, middle, or low rank, based on our previous observation). We did not analyze data by month because fruit did not vary in abundance during each year’s two-month window^50^. We set several seed dispersal parameters based on previous studies^29,51^. First we calculated the frequency of seed occurrence, which was defined as the number of fecal samples containing seeds, for each year and dominance rank of macaques for each plant species. Since we did not collect fecal samples in equal quantities across the two years and dominance rank of macaques, we calculated seed appearance ratios (the number of dispersal events divided by the number of fecal samples examined). Secondarily, we calculated seed appearance ratios for each year and dominance rank of macaques, for all plant species combined, and for each species separately. For each fecal sample in which seeds appeared, we then recorded the number of seeds in the sample as the quantitative index of seed dispersal. We also recorded the seed intact ratio (the ratio of seeds with no apparent physical damage after gut passage) as another qualitative index of the efficacy of seed dispersal^29^, and the seed species richness as an index of plant diversity.

### Frugivory

Here we use data collected from early October to late November in 2004 (38 days) and 2005 (33 days), which is a subset of our previous study^27^. The total observation time was 479 hr (276 hr in 2004 and 223 hr in 2005). We recorded activity patterns every 1-minute using instantaneous sampling (3–5 hours per single session). We classified activity into feeding (including picking up, processing, and chewing at one location) and others (moving, resting, social grooming, and others such as drinking, fighting, and alarm calling). If the focal animals were feeding, we classified food items into fleshy fruits, nuts, or others (leaves, herbaceous plants, fungi, animal materials, soil and unidentified materials). Details of the observation are published in a previous study^27^. For each food category, we defined the percentage of feeding on it as the percentage of instantaneous sampling points consuming the food item relative to all sampling points associated with feeding. In this study, we treated the percentage of the fleshy fruit in the diet as an index of dispersal propagules^7^.

### Statistical analysis

We performed chi-square tests for independence to examine inter-annual and inter-rank difference in the seed appearance ratios for each fleshy fruit species separately, and for all the species combined. To evaluate both inter-annual and inter-rank differences in the number of seeds within single fecal samples we employed generalized linear mixed models (GLMM) with count data (with the Poisson error distribution). We included the identity of each animal as a random effect in this and following GLMM models. Only fecal samples containing seeds were included in this analysis. The response variable was the total number of seeds found in each fecal sample, and explanatory variables were year, dominance ranks, and their interaction. In order to test the inter-annual and inter-rank differences in the seed intact ratio, we fitted another GLMM with the negative binomial error family, due to overdispersion of the seed intact ratio. The response variable in this case was the number of intact seeds in each fecal sample (the overall number of seeds was treated as an offset term). To analyze the seed species richness in fecal samples, we again ran a GLMM (Poisson error family) with the number of seed species as the response variable and the fixed effects were the same as those in the first and second GLMM models. Finally, to examine the effects of year, dominance rank of macaques, and their interaction on the percentage of the total feeding, nut feeding, and fleshy fruit feeding, we used another GLMM with the negative binomial error family. The response variable in this case was time spent on a given activity (represented by the number of instantaneous samples). The overall number of instantaneous samples was treated as an offset term). We performed the GLMMs using the “glmmML”, “aod”, and “nlmn” packages in the statistical software R.3.3.2^52^. The significance levels of these analyses in this study were set α = 0.05.
